# Following the footsteps of Burmeister's leaf frog (*Phyllomedusa burmeisteri*) in the Atlantic forest of Brazil

**DOI:** 10.1038/s41598-023-43491-2

**Published:** 2023-10-04

**Authors:** Daniela Pareja-Mejía, Júlia Benevides, Lidiane Gomes, Edvaldo Moreira Da Silva Neto, Vinícius Queiroz Menezes, Rafaella Silva Roseno, Amanda Sabino Martins, Mirco Solé

**Affiliations:** 1 Graduate Program in Zoology, Universidade Estadual de Santa Cruz, Ilhéus, Bahia Brazil; 2 Department of Biological Sciences, Tropical Herpetology Laboratory, Universidade Estadual de Santa Cruz, Ilhéus, Bahia Brazil; 3 Graduate Program in Ecology and Biodiversity Conservation, Universidade Estadual de Santa Cruz, Ilhéus, Bahia Brazil; 4 Herpetology Section, Zoologisches Forschungsmuseum Alexander Koenig, Bonn, Germany

**Keywords:** Ecology, Zoology

## Abstract

Amphibians are organisms which mainly have a biphasic life cycle. When at the larval stage, their habitat is generally aquatic, and when adults, they become terrestrial. Pond-breeding amphibians are sensitive to some disturbances in their environment which lead to the decline of a population. The interactions between the species and their environment are performed through movement. Movement ecology combines and explains the movement data of organisms with biotic and abiotic factors and because of this, knowing the movement of these creatures is of great ecological importance. We used the spool-and-line methodology in individuals of the treefrog *Phyllomedusa burmeisteri*, at the Reserva Ecologica Michelin, located in the southern region of Bahia in Brazil to study their movement patterns in different environments inside and outside of the forest. We monitored 19 *P. burmeisteri* individuals that presented a mean total movement distance of 2160.76 cm (S.D. 1152.42). We found no significant difference in the trajectories of individuals in forested and open areas, as well as a positive relationship between the distances individuals moved and their weight only in forested areas. no relationship between distance and weight of these individuals. We observed that individuals followed non-linear paths and the number of steps from one place to another varied among individuals. Our movement ecology data allows us to answer questions about short-term movement patterns of *Phyllomedusa burmeisteri*. This is the first step to start understanding the spatial cognition of treefrogs from the Atlantic Forest and to fill gaps about life habits of these frogs. Information on the movement patterns of a species, as well as its home range can help to create conservation strategies, regarding the creation or delimitation of protected areas, for example.

## Introduction

Permanent and temporary ponds are essential breeding sites for pond-breeding amphibians due to their biphasic life cycle, composed of an aquatic larval stage (i.e., tadpole) and a terrestrial (i.e., juvenile and adult) stage^1^. Ponds are dynamic sites with food availability, suitable climatic and water conditions, and the possibility of gene exchange with migrating individuals. However, breeding ponds can suffer from disturbances such as habitat fragmentation^2^. The daily movement and dispersal ability of a species are factors that can delimit its persistence in the face of these and other biotic and abiotic disturbances^1,3^. In this context, describing the movement of amphibians has become a key ecological aspect to drive species conservation strategies^4^ since understanding movement enables to identify threats, such as the loss of important sites or barriers to movement, for example.

Identifying the movement of individuals translates their interactions with the environment on a spatial and temporal scale, which can be influenced by several factors such as landscape structure and resource availability and predictability^5–8^. Such movement behavior influences the organism as it affects its fitness and development, increasing or decreasing its survival, adaptation, and favoring in natural selection processes. On the population scale, movement influences gene flow, population persistence, metapopulation dynamics, and species distribution^5,9^. Movement in amphibians provides information regarding migration, dispersal, homing activity, home range, and site selection for reproduction, among others^1^. Research focused on animal movement is necessary to get to know and understand species’ ecology. Monitoring amphibian movement has always been a challenge, especially because the effectiveness of the tracking mode depends not only on the environment and habitat, but also on the morphology of the individuals such as the size and shape of the body, which could also affect the selection of tracking devices and how they are attached to the individual^10^.

Among the methods already used to follow the movement of anurans, the most successful and used are radio telemetry, fluorescent powder, and spool-and-line. Radio telemetry is the most common method but may present difficulties when used with small animals^11^, since an animal should not carry more than 10% of its body mass^12^. Another limitation of radio telemetry is that it only offers isolated points and not a detailed trajectory. The spool-and-line methodology may be considered the most viable for some species. This is due to the low cost of equipment, easy adaptation of the size of the line and the device to the weight of the individual (which consequently makes it less invasive), and a thorough mapping of the distance, height, and angulation of the steps in the organisms being tracked^10^.

The anuran genus *Phyllomedusa* has a peculiar method of locomotion: instead of jumping, these animals walk slowly on their legs, clinging to habitat surfaces such as branches in the vegetation^13^. To understand and describe the daily and total movement patterns of *Phyllomedusa burmeisteri* in fragments of the Atlantic Forest of Brazil, we applied the spool-and-line methodology, previously tested by Mejía and collaborators^10^. This method is advantageous for this species since it allows fluidity in the movement of individuals who can be found on vegetation and occasionally on the ground. We aimed to (i) describe the movement patterns of *Phyllomedusa burmeisteri* and estimate the distances they move daily and for four days, (ii) observe if there is a relationship between movement and weight of individuals of *P. burmeisteri*, and (iii) observe if organisms from ponds inside the forest have different movement patterns when compared to individuals in ponds outside forested areas.

## Methods

### Ethics statement

We had approval to obtain and/or observe specimens in the field from the Ministério do Meio Ambiente—MMA, Instituto Chico Mendes de Conservação da Biodiversidade—ICMBio and Sistema de Autorização e Informação em Biodiversidade—SISBIO (authorization n 73371-3). The project "Movement ecology of Neotropical anurans" (010/20) which this research is part of was approved by the Ethics commission of Universidade Estadual de Santa Cruz and all methods were followed in accordance with the guidelines and regulations of the Ministério do Meio Ambiente—MMA in Brazil.

### Study site

**Figure 1:**
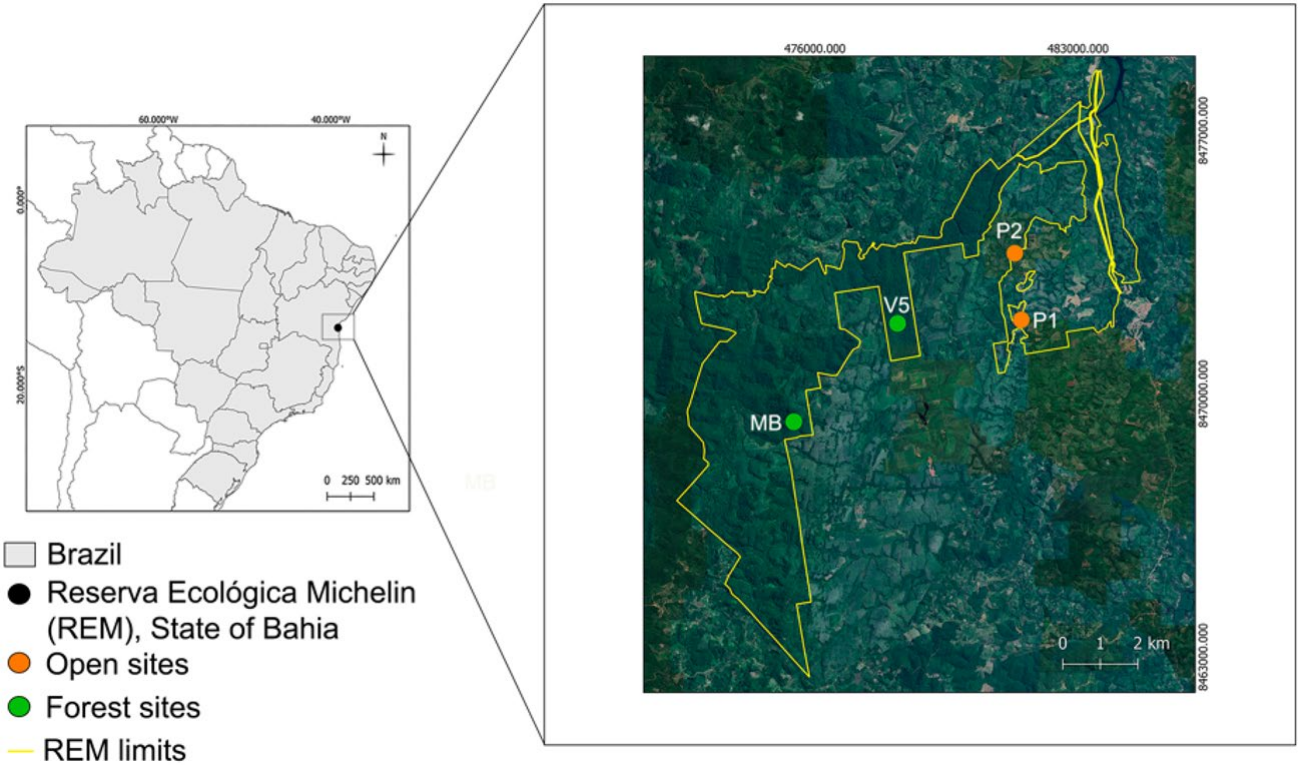
Map of the Reserva Ecológica Michelin, Bahia State, northeastern Brazil. The circles represent the four study sites inside of the Reserve (MB: Mata Boa, V5: Vila 5, P1: pond 1, P2: pond 2). The map was created using the EPSG: 32724 WGS 84/UTM zone 24S datum. The satellite image used was from the year 2022. Figure created by LG.

We carried out our research in the Reserva Ecológica Michelin (REM) (Fig. 1), located between the municipalities of Igrapiuna and Ituberá (24,481,896 mE, 8,474,140 mS), in southern Bahia, northeastern Brazil. The REM has a total of 3800 ha of which 1800 ha are perennial floodplain forest and is classified as a Dense Ombrophilous Lowland Forest by Veloso et al.^14^. The topography varies between elevations of 40–586 m. The average annual precipitation is 2000 mm, with no defined dry season and daily temperatures oscillate between 21 and 30 °C^14^. It is embedded in a regional landscape (1000 km^2^) that retains 40% forest cover and a high diversity of agroforestry systems with more than 60 planted tree crops^15^. We sampled ponds inside forest fragments (Vila 5, N = 1; Mata Boa, N = 1), water courses in forest fragments (Vila 5, N = 1); and temporary and permanent ponds in open areas (Open sites: P1, and P2; N = 2) (Fig. 1). The ponds located in the forest fragments are part of the early secondary forest, characterized by dense continuous vegetation and spaced taller trees. Ponds are centralized, completely covered by vegetation on the sides, and distant from the main forest trails. As for open area ponds, they are surrounded by scarce vegetation, mostly smaller in height and diameter than that found in the forest and are close (approximately five to 10 m) to areas with traffic of cars and trucks. The ponds in open areas are located near rubber plantations.

### Study species

The species *Phyllomedusa burmeisteri* belongs to the family Phyllomedusidae and is grouped in the *P. burmeisteri* complex which includes five species: *P. bahiana*, *P. burmeisteri*, *P. distincta*, *P. iheringii* and *P. tetraploidea.* Several authors^13,16–18^ have failed to separate *P. bahiana* and *P. burmeisteri* but none have taken any taxonomic approach. De Andrade et al.^19^ performed an acoustic and morphometric evaluation of the geographical distribution of the species *Phyllomedusa burmeisteri*; but were unable to differentiate traits between *P. burmeisteri* and *P. bahiana*. Therefore, we decided to also call our studied individuals *Phyllomedusa burmeisteri* as it is the older name. *P. burmeisteri* is a medium-sized species (SVL = 53–70 mm) with arboreal and nocturnal habits, where males can be found vocalizing over the marginal vegetation of permanent and temporary ponds, as well as in streams^20^. It is distributed in eastern Brazil in areas of Atlantic Forest, with records in the states of Minas Gerais, Bahia, and São Paulo^21,22^. Some aspects of the biology and life habits of *P. burmeisteri* are known, such as its wide repertory of calls, territorialism, and site fidelity^23,24^. However, being a species found in forests and well-preserved environments, but also tolerating environments disturbed by human actions, nothing is known about its ability to move, perform or even if there is a preference between these different habitats. In addition, it is a species that can be found in the REM throughout the year and that occupies both pools inside and outside the forest, thus we believe its plasticity to different environments allows it to be a suitable model for our study.

### Data collection

#### Preparation of tracking devices

**Figure 2:**
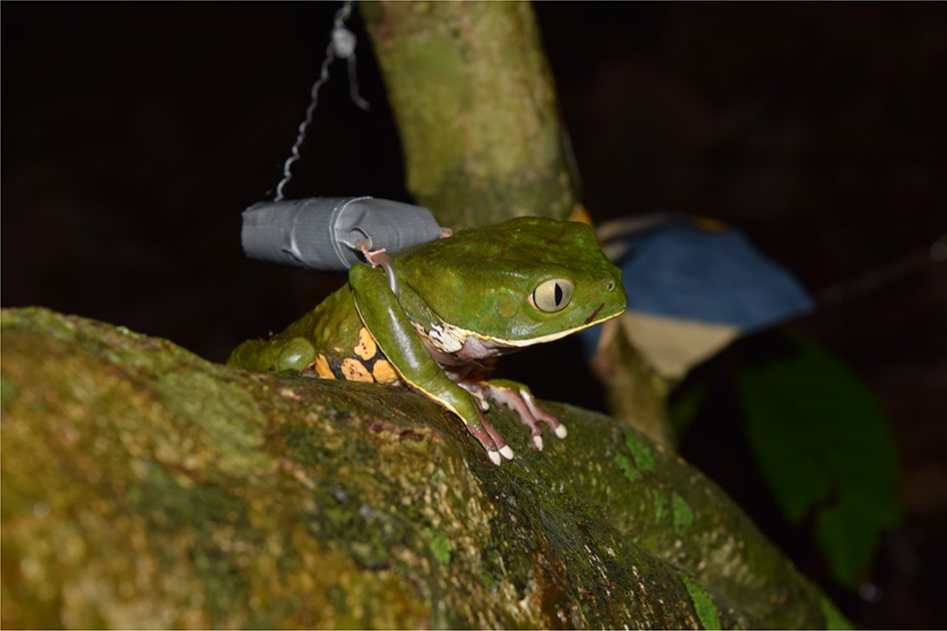
*Phyllomedusa burmeisteri* individual with the tracking device. The spool-and-line device was attached to its back and the individual was then released at its capture site.

All monitoring devices were constructed in the field at the time of capture to better adapt to the size of the individual and reduce the likelihood of physical damage. To do this, we optimized time by adjusting bobbins in different weights so that they could fit randomly sized individuals. Each spool acquired from the factory has a total length of 250 m and weighs 4.8 g. We prepared several bobbins weighing on average 10% of the average weight of *Phyllomedusa burmeisteri*. When we found an individual, we weighed it and chose one of the bobbins that matched its weight respecting the 10% rule^12^. Only this part of the device was developed outside of the field. The materials and prototype used were suggested by Mejía et al.^10^. The device consists of four pieces, with two circles for the front legs and two linear parts for the dorsal and ventral region of the neck. We used the 23G butterfly needle disposable microtube (Tianjin Hanaco Xingda Medical Product CO LTD) for the four pieces, joined by a 100% cotton thread (Kite Thread—Coats Current) which ensured the mobility of the individual and prevented the breakage of the pieces throughout the monitoring. The spool was made of nylon thread (HILTEX IND. E COM. DE FIOS LTDA), wrapped in plastic with one end free to allow the thread to unwind. Silver tape (3 M Scotch) was used to protect the spool from humidity and water absorption, before attaching the device to the individual (Fig. 2).

#### Tracking and data collection

We conducted our study in August and November 2021 and January 2022. The period of *Phyllomedusa burmeisteri* collection and monitoring activities totaled 40 days, 15 in August/2021, 20 in November/2021 and five in January/2022. At the end of this study, we can account for approximately 152 h of sampling effort in the field (2 h per day/per individual), and 40 h for preparation and organization of materials (1 h per day). Our field schedule started in the two ponds outside the forest area (P1 and P2) that are located near the Reserve’s headquarters. In these ponds we divided the team into two groups: the first group was responsible for the active search and collection of new individuals, and the second group was responsible for monitoring and recording information for individuals that had received the tracking device the day before. We then continued to the two ponds inside the forest areas (V 5/MB) where the same methodology was performed.

Individuals were found by visual search at night near ponds and flooded areas. Once an individual was located, coordinates were taken, and we marked the exact place where we found the frog with marking tape. We then weighed the frog and took a picture of the ventral pattern of each individual. We used a photo ID methodology^23^ and created a photo library to avoid recaptures. We attached the “backpack” device on each individual and then also attached the spool-and-line which weighed less than 10% of the individual’s weight^12^. After the device was attached, we released the individual frog at night on the same day it was captured and at its original capture site. We then observed its movements and behavior for ten minutes to ensure that it was able to move normally before walking away.

**Figure 3:**
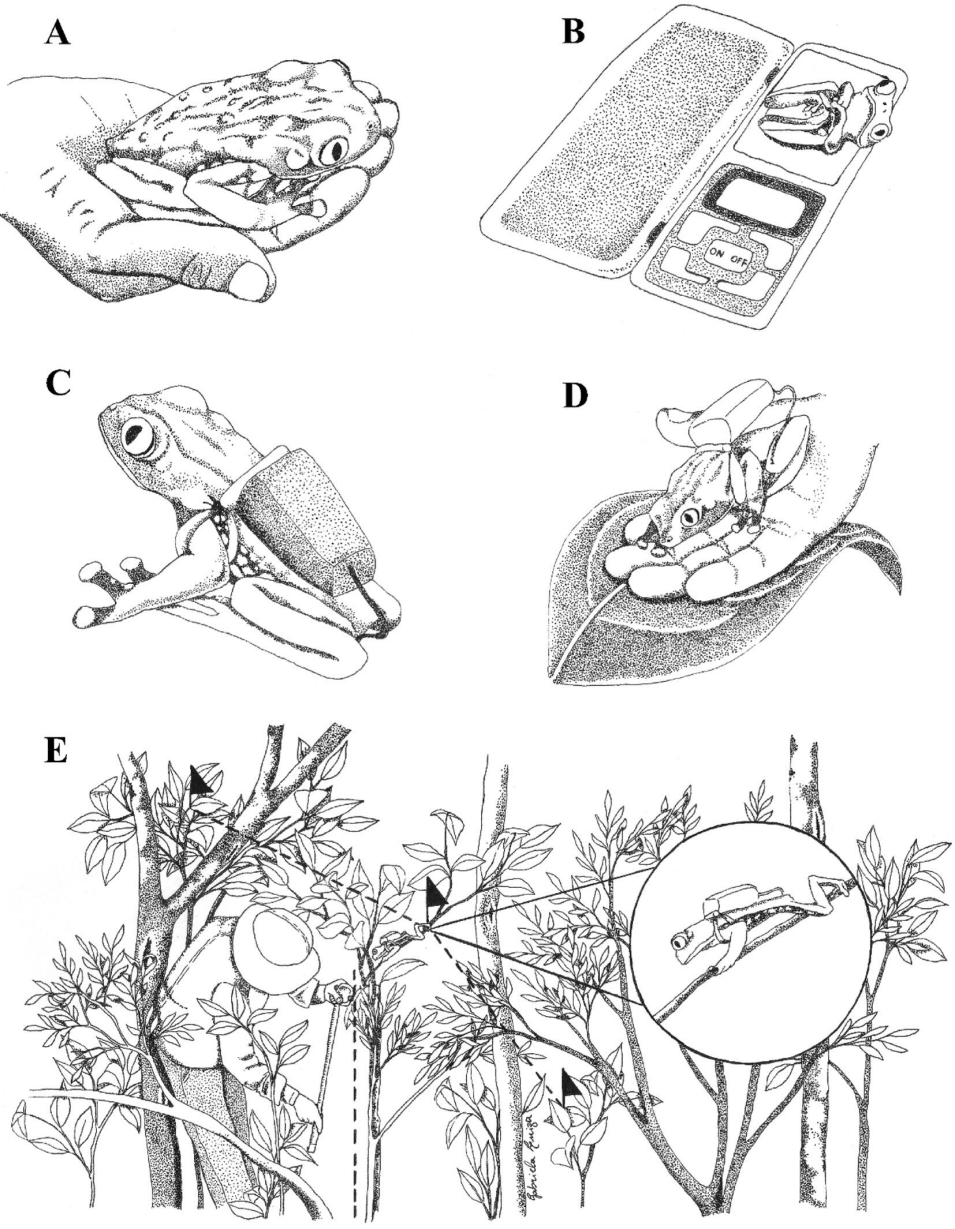
Protocol for monitoring treefrogs in the field. (**A**) Capture of the individual and data collection, (**B**) Weight of the individual, (**C**) Attachment of spool-and-line device, (**D**) Release and observation, (**E**) Daily measurements of distances, heights, and angles. Dotted line simulating the distance (perpendicular line between flags) and height (vertical line) measurements. Black flags represent the signaling of steps. Illustrator: Gabriela Luiza de Deus.

According to Mejía et al. and Richards et al.^10,12^ the size of the threads usually used for *Phyllomedusa* individuals is long enough for them to move and to be tracked daily (a 0.28 g bobbin holds approximately 10 m of thread), so we monitored each individual once a day over a four-day period. Each day we followed the trail of thread until we found the individual. At this point, we measured the thread’s path marked by steps and stops, the height from ground where the individual was found, and coordinates (i.e. latitude and longitude) were recorded. We defined a step as the distance between two points when the individual made a turn, i.e. when the turning angle changed. We also checked the device and width of the bobbin and assessed the frog for any injuries. If we observed that the length of the remaining thread would not be sufficient to track the movement over the next 24-h period, we replaced the bobbin. We recorded information about the individual’s activity (if it was resting or moving), and any notes on behavior observed during the encounter. After the fourth day of each individual monitoring, the “backpacks” were taken off and all signs were removed from their marking locations (e.g., branches, soil, and vegetation). Figure 3 compiles our protocol in the field.

### Data analysis

We performed two *t* tests, firstly to investigate whether total movement distance was different between individuals in ponds inside the forest and individuals in ponds outside forested areas. In this test y is the movement total distance of each individual and x the type of pond (located in the forest or outside the forest). Second, we carried out a t-test to evaluate whether the maximum height traveled differed between individuals in both areas, with y being the maximum height reached by each individual and x being the ponds (forest or outside forest). We also tested whether there is a relationship between the movement total distance and the weight of individuals through an analysis of a Generalized Linear Model (GLM), using the family = "Gamma". The model assessed distance by weight and area, and subsequently, we constructed distance by weight models for forested and open areas (Additional Files 1, 2, 3 and 4). We used the “ggplot2” package^24^ and the “plot3D” package^25^ in R software to create the figures. To determine whether the motions followed an orientation preference, Kuiper’s test of uniformity was used for each rose diagram with the R software package “CircStats”^26^. The analyses and figures were performed and created in the R environment^27^.

### Ethics approval and consent to participate

Individuals were collected under the permanent permit n 73371-1 issued to Mirco Solé by Ministério do Meio Ambiente—MMA, Instituto Chico Mendes de Conservação da Biodiversidade—ICMBio and Sistema de Autorização e Informação em Biodiversidade—SISBIO. The project "Movement ecology of Neotropical anurans" (010/20) which this research is part of was approved by the Ethics commission of Universidade Estadual de Santa Cruz.

## Results

We successfully tracked a total of 19 *Phyllomedusa burmeisteri* individuals with the spool-and-line methodology for a period of four days each. We observed normal behavior in individuals equipped with the tracking device since they were able to call, to move through aquatic environments, to climb trees and even cross and shelter in herbaceous microhabitats.

**Figure 4:**
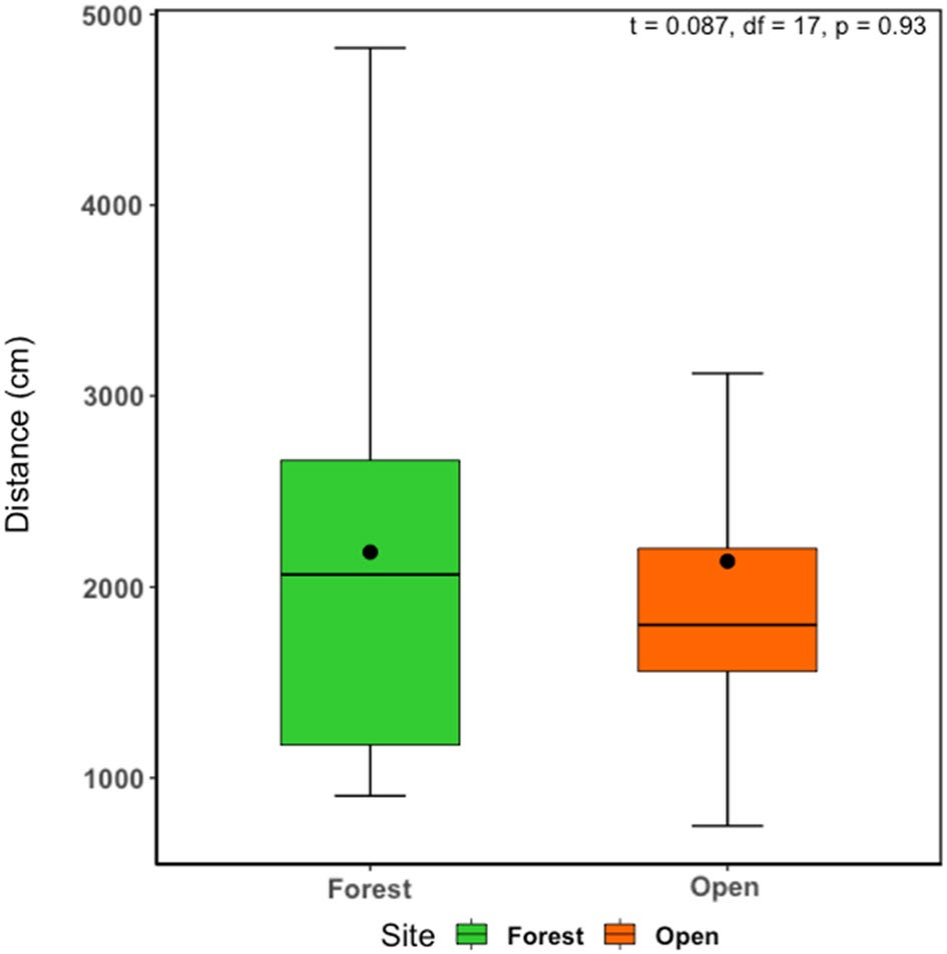
Boxplot showing the difference in total distances traveled by individuals in ponds inside the forest and ponds in open areas.

The mean movement total distance for all individuals recorded using spool tracking was 2160.76 cm (SD 1152.42). We found no significant differences between individuals in the forested area and the open area (t = 0.087, df = 17, p = 0.93) (Fig. 4). Table 1 shows the weights of individuals, and their devices, the true distance traveled for four days, the daily distances moved and the height from the ground from field observations.

**Table 1:** Information on the individuals tracked [sex, weight of the individual (W), weight of the device (WD), total distance moved (TD), mean daily distance (DD), total steps (TS), maximum height from the ground (H), and monitoring site].

Id	Sex	W (g)	WD (g)	TD (cm)	DD (cm)	TS	H (cm)	Site
PB1P2	Male	14.28	1.4	2203	71.1	30	80	Open
PB3P2	Male	14.05	1.39	2142	94.8	27	50	Open
PB1P1	Male	13.64	1.25	1802	129.9	19	50	Open
PB2P1	Male	12.52	1.22	3118	79.3	24	348	Open
PB3P1	Female	20.5	1.9	4700	123.7	38	240	Open
PB4P1	Male	15.65	1.4	1558	82	19	133	Open
PB5P1	Male	14.88	1.44	748	53.4	14	158	Open
PB6P1	Male	13.52	1.25	1702	85.1	20	274	Open
PB7P1	Male	17.37	1.5	1248.5	65.7	19	183	Open
PB1MB	Male	21.72	2.15	2703	107.8	31	400	Forest
PB2MB	Female	40.77	3.96	4824	383.3	34	638	Forest
PB6MB	Male	17	1.6	906	90.1	12	54	Forest
PB7MB	Male	21.4	2	1000	81.9	12	138	Forest
PB8MB	Male	18	1.6	1021	141.9	9	40	Forest
PB10MB	Female	22.4	2.1	1832	60.3	17	440	Forest
PB11MB	Male	19.5	1.9	2300	110	6	1200	Forest
PB1V5	Male	20.2	2	2540	75.5	30	175	Forest
PB3V5	Male	19.6	1.85	1627	83.3	27	185	Forest
PB4V5	Male	17.17	1.68	3080	102.1	28	460	Forest

**Figure 5:**
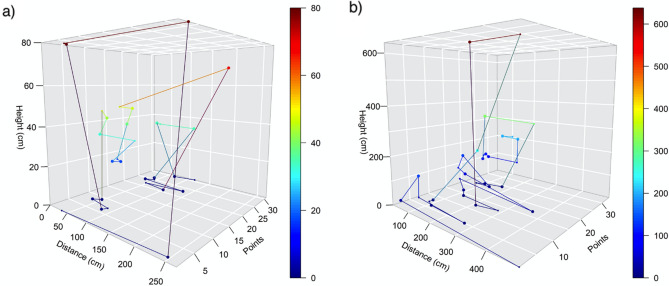
Movement of two individuals during the four days of monitoring, showing three dimensions: distances traveled, height from ground and each point measured; (**a**) individual from pond in open area (PB1P2); (**b**) individual from pond inside the forest (PB2MB). *The organisms were chosen randomly just to show the three dimensions of movement patterns of *Phyllomedusa burmeisteri.*

**Figure 6:**
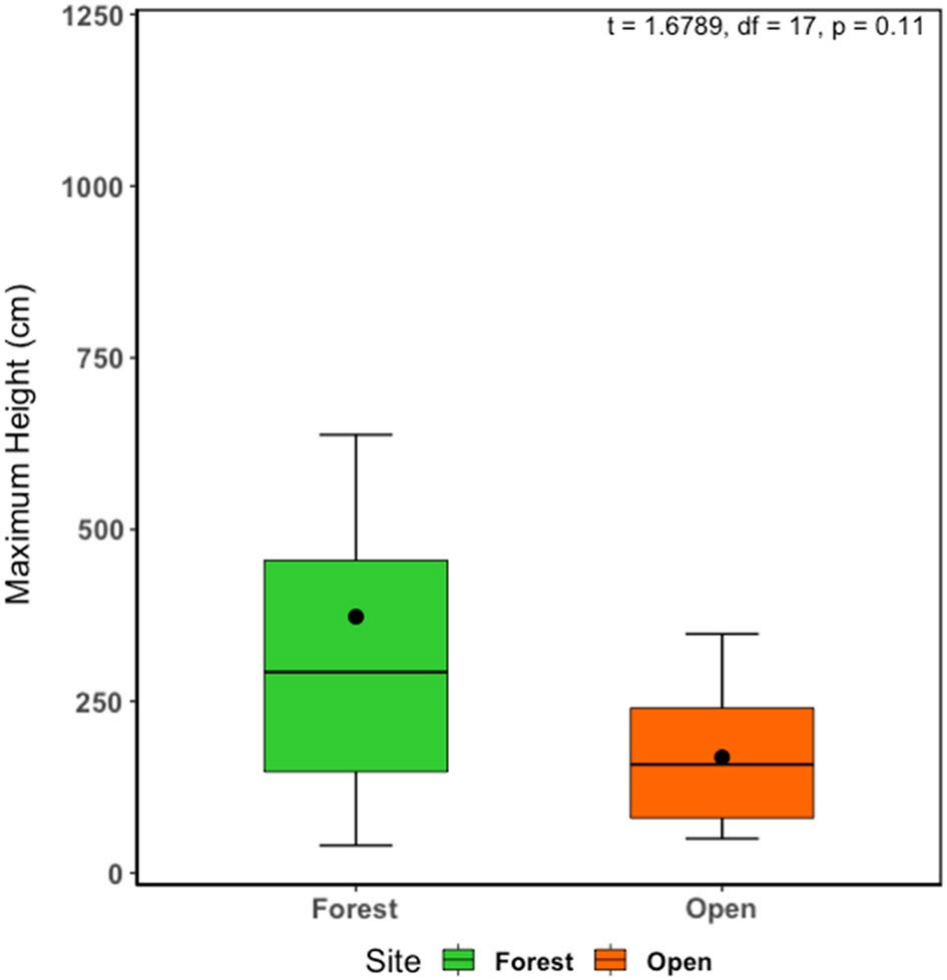
Boxplot showing the difference in total heights reached by individuals in ponds inside the forest and ponds in open areas.

In general, individuals did not move linearly between capture/recapture sites, but instead moved erratically alternating between horizontal and vertical movements. We observed that heights reached at forested areas (average 373.00 ± 331.01) were higher than the ones on open areas (average 168.44 ± 97.72) (t = 1.6789, df = 17, p = 0.11) (Figs. 5 and 6, Additional files 5 and 6).

**Figure 7:**
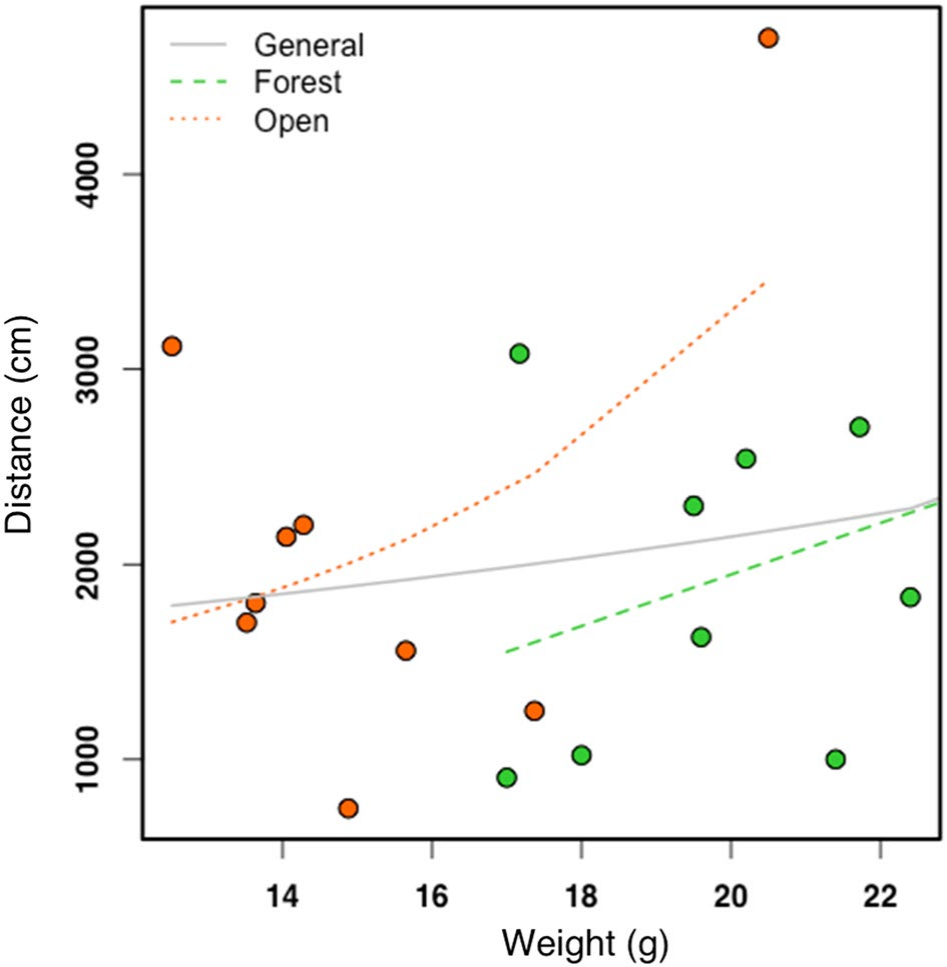
Relationship between distance traveled and weight of the individuals from ponds inside the forest and ponds in open areas.

We observed a positive relationship between the distances individuals moved and their weight [F(1,17) = 6.933, p = 0.01745] (Fig. 7). This relationship also occurred in the forested area [F(1,8) = 11.06, p = 0.01], where individuals of greater weight traveled a greater distance in this environment. And we didn't observe any effect of weight in the open area [F(1,7) = 1.84, p = 0.2171]. The heaviest individual (PB2MB, m = 40.77 g) was the one that moved the longest distance (D = 4824 cm), however we also observed that individuals half of its size were able to complete a similar distance (PB3P1, m = 20.5 g, D = 4700 cm).

**Figure 8:**
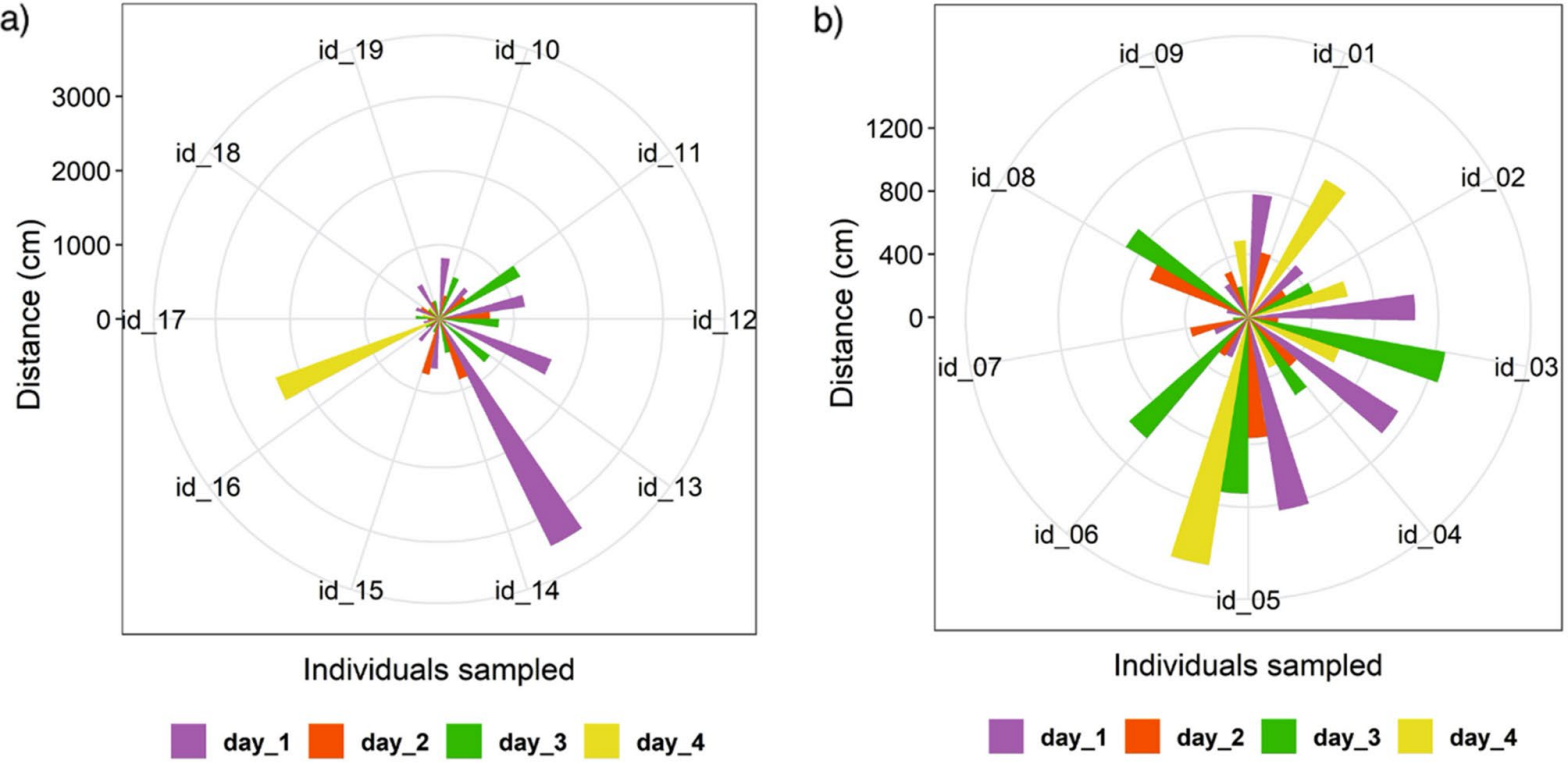
Distances traveled by each individual in the four monitored days; (**a**) individuals from forest ponds; (**b**) individuals from ponds outside the forest.

Overall, we observed that daily activity patterns varied from one day to another. Four individuals were more active during the first two days and then stopped moving (PB4P1, PB6P1, PB3V5 and PB8MB), whereas some animals moved very little during the first days and then covered longer distances (Fig. 8). The number of steps made by individuals also differed, however we didn’t observe significant differences between individuals inside the forest and in open areas.

**Figure 9:**
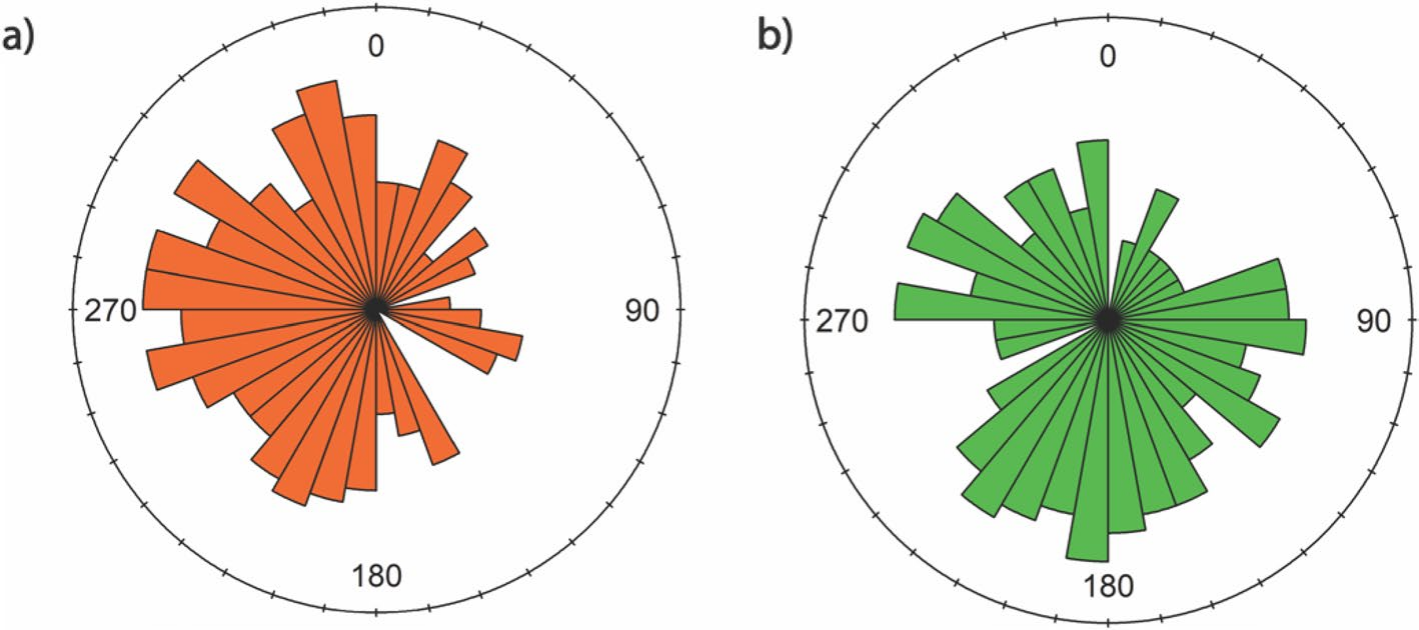
Rose diagram showing the directionality of movement of individuals. (**a**) individuals from forest ponds; (**b**) individuals from ponds outside the forest.

Individuals from open areas moved more frequently with orientation following a uniform path to the north, west and south, and less to the east (D = 3.46490, *p* ≤ 0.01, N = 174) (Fig. 9a). On the other hand, individuals from forested areas moved more frequently with a preferential orientation towards the west, south and east, and less towards the north (D = 2.2888, *p* ≤ 0.01, N = 146) (Fig. 9b).

## Discussion

Movement ecology is gaining momentum in herpetology and many aspects of the ecology and behavior of herpetofauna are being unveiled by this. However, there are still only few studies conducted in Brazil^10,28^ and this work aimed to fill in the gap information on the movement patterns of *Phyllomedusa burmeisteri* in the Atlantic Forest. In this study, we know the patterns of movement of *P. burmeisteri*, as well as the distances individuals moved inside or outside of the forest. However, forest individuals’ moved greater heights than individuals from outside the forest. We observed that the pattern of movement was not constant among the individuals during the four days of monitoring, with days that individuals moved and others that they remained in the same location. In addition, orientation varied between individuals from forest and open areas, with individuals from forest areas moving more towards the north, east and south and individuals from outside the forest areas moving north, west, and south.

We confirmed that the attachment method proposed by Mejía et al.^10^ is effective and allows the movement of *P. burmeisteri* and its monitoring for short periods of time. Likewise, the spool-and-line is an adequate methodology for the observation of more accurate details of the individual’s movements^29^ and could be used in other frog species as well. We showed that even if *Phyllomedusa burmeisteri* show slower and delicate movements, their daily displacements were significant and comparable to those of other treefrogs. Previous short-term studies of amphibian movement have described varying movement rates, with individuals moving on average 5 m or less per day^11,28,31–33^; however, most of these studies have used linear distance estimates and haven’t taken the real estimates, including information regarding the turning angles, into account.

We did not find significant differences between the distances individuals moved inside or outside of the forest; however, we were able to establish the movement patterns of *Phyllomedusa burmeisteri* in these two types of areas. We observed that individuals didn’t move in a linear way and interspersed with vertical and horizontal movements. We found that both vertical and horizontal movements are important for this species since *P. burmeisteri* uses the three-dimensional structure that tropical rainforests present. Even though this species is mostly found in tree branches, it also uses microhabitats such as low vegetation (herbs and logs), as well as medium and higher vegetation and aquatic environments. When comparing with other tree frogs, Moser et al.^30^ observed *Boana marginata* using microhabitats with lower vegetation densities than *B. bischoffi*, being more exposed in the environment. In general, our individuals of open areas preferred lower vegetation, and were frequently recorded at lower heights, including on the substrate, even if they had large trees and medium vegetation at their disposal. This can perhaps be explained by the fact that it was not their breeding period (in general the individuals were not calling) and high exposure is one of males’ behavioral strategies to win sexual partners^31^. These erratic movements have also been observed in *Phyllomedusa trinitatis*^32^ and *Leptodactylus labyrinthicus*^33^. This may be related to migration and dispersal movements by amphibians in which they do not necessarily use the same routes and prefer some routes more than others^8^.

We observed that most of the individuals moved during the four days, however we could see that some moved more during the first two days and then we encountered them in the same place for the following two days. This was also observed in the study by Moser et al.^30^ where they found several individuals at their capture sites for several consecutive days. It is likely that this variation occurs due to the selection males perform in the search for their calling sites. When they found their “optimal” sites they would tend to perform little to no displacement at all^30^. The lack of movement can also be considered a strategy to save energy for the breeding season^36–38^ as studied in other species.

In the case of pond-breeding amphibians, it is known that the pond is the essential site around which the population is organized^39^. In all cases, the *P. burmeisteri* we monitored were found around the ponds, but none were found at the exact edge of the ponds. The movement patterns we observed were therefore from trees or branches in the surroundings towards the ponds or simply between trees close to them. This could also be evidenced by the orientation of the movements, in the case of open areas, the ponds were located west, and the individuals moved more frequently with orientation following a uniform path to the west, north, and south, and less to the east. On the other hand, ponds in forested areas were located south and individuals from forested areas moved more frequently with a preferential orientation towards the southwest, and east, and less towards the north. Directionality of movements can be an important characteristic that helps to explain orientation. In fact, beaconing towards an odor-emanating pond or piloting towards forest edges or other visual, olfactory, or acoustic landmarks are common orientation mechanisms, and this could be potentially useful to identify breeding sites or suitable summer habitats in unknown areas as well^40,41^.

Several studies^28,33–35^ have compared the movements between males and females and found no significant differences even though males are supposed to move less than females, especially during breeding seasons. We couldn't make this sex comparison due to the low number of females found during our monitoring period, but we were able to find significant differences between the distances and the individuals’ weight in forested areas. We also observed that the two females of our study moved considerable distances during the four days compared to the rest of the individuals. However, we agree with de Oliveira et al.^28^ that the low variation in body sizes and the number of individuals found during our study period did not allow us to observe this and other types of variations in our analysis.

Overall, our study showed a difference in the movements of *P. burmesteri*, individuals from the area inside the forest had higher vertical movement patterns than individuals from outside the forest, but we could not infer whether this change in movement pattern can be caused by a more restricted use of space by individuals from areas outside the forest. Moreover, as we did not sample the structural aspects of the forest and the area outside the forest, it was not possible to make a comparison between the use of vertical space and the number and height of trees in the two areas. We believe that future studies can carry out more detailed sampling of the structural aspects of the areas to make this comparison of the vertical movement pattern. Furthermore, the movement pattern may vary according to the diet of individuals, if the food supply is different in the two environments, but diet studies of this species are not found in the literature. Our work was pioneer in testing the movement pattern for an arboreal species, but we realized that there are still many questions to be solved about the life history of the species, and inserting relevant information on structural and microclimatic aspects of the sampling areas, which should help in a better understanding of the movement of *P. burmesteri*, and thus we can use movement patterns to assist in the conservation of arboreal species. Integrating amphibian movement and conservation measures is often difficult due to the lack of studies on the subject and their depth^4^. Our study has important data on the movement patterns of a species that we consider has a high plasticity with respect to others and is able to be present in both fragmented areas and in secondary forests of the REM. Studies such as this can contribute to the creation of potential conservation strategies such as identify and protect important features of the habitats (foraging habitat, breeding areas, refugia) of arboreal species like *Phyllomedusa burmeisteri*.

## Conclusions

Our data provides significant information on the movement behavior of *Phyllomedusa burmeisteri*, which is a species little studied to date. We provided consistent data on their mobility in areas inside and outside of the forest. Our study revealed that the movement patterns of *P. burmeisteri* were not constant across individuals during the monitoring period. The orientation of movement varied between individuals from forested areas and open areas as well. This suggests a dynamic and non-linear movement behavior, indicating potential variation in factors influencing movement, such as breeding site selection or energy conservation. These data are important not only in a descriptive context of their natural history and ecology but also to reveal information that may be important for the conservation and management of tree frog species. Understanding movement patterns and habitat preferences can aid in the identification and protection of crucial features of their habitats, such as foraging areas, breeding sites, and refugia. We will conduct longer periods of monitoring movements, and in different seasons to gather more data and to allow the description of the home range of this and other species of tree frogs. This is the first step for a long-term study of spatial cognition in tropical forests.

### Supplementary Information

Supplementary Information.

## Data Availability

The data are available upon request from the authors. Information or data request should be done to the corresponding author Daniela Pareja-Mejía.

## Abbreviations

REM: Reserva Ecologica Michelin
ID: Identification
