# Supplementary material for: Following the footsteps of Burmeister's leaf frog (Phyllomedusa burmeisteri) in the Atlantic forest of Brazil
Source: Sci Rep. 2023 Oct 4;13:16698. doi: 10.1038/s41598-023-43491-2 (PMC10550930; doi:10.1038/s41598-023-43491-2)
Supplement: Supplementary file 1 — Supplementary Information. [file 41598_2023_43491_MOESM1_ESM.pdf]

a) b)

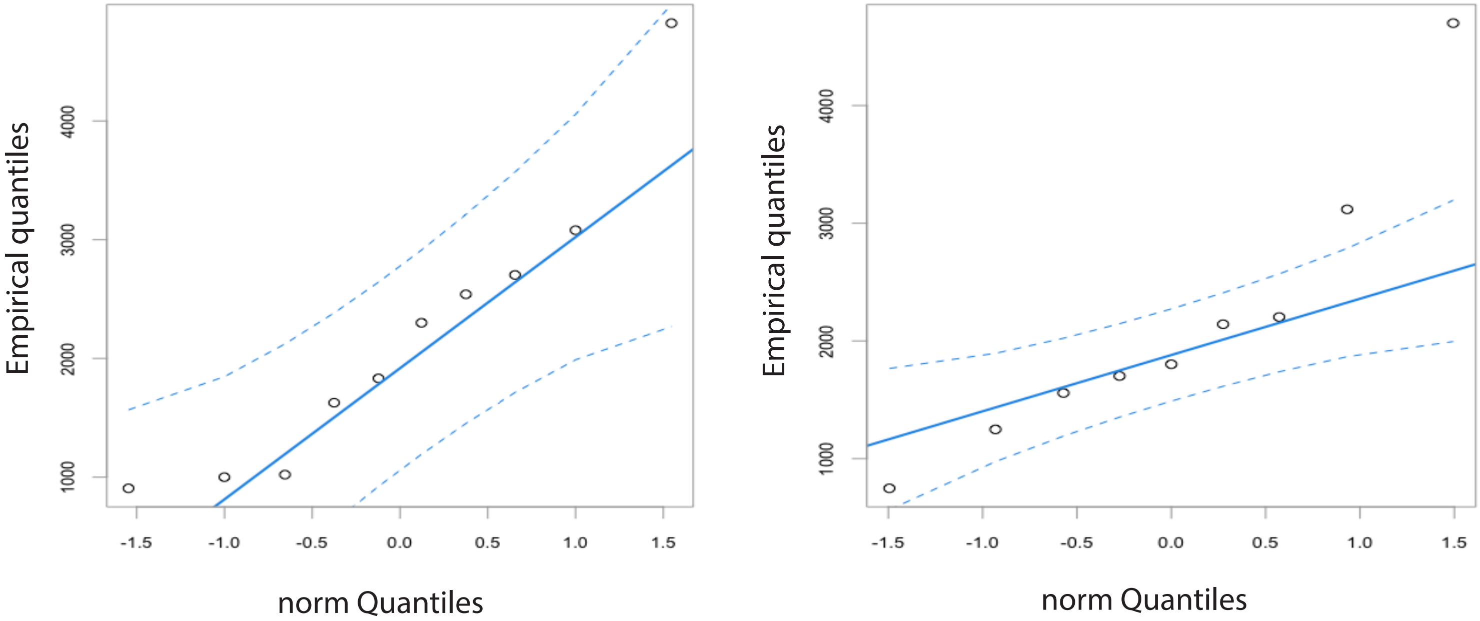

Additional File 1. Normal Q-Q Plot for t-test for total distance traveled by area a)forest and b) open

a) b)

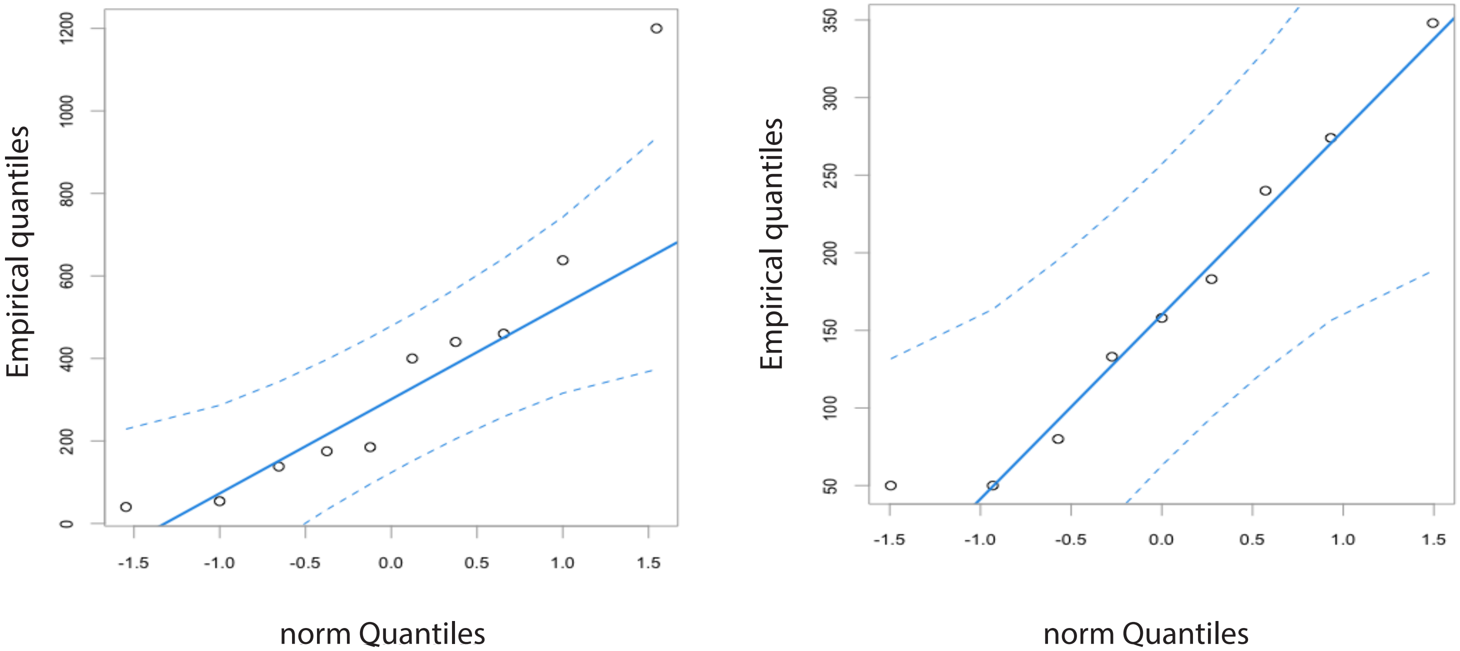

Additional File 2. Normal Q-Q Plot for t-test for maximum height in a) forest and b) open areas

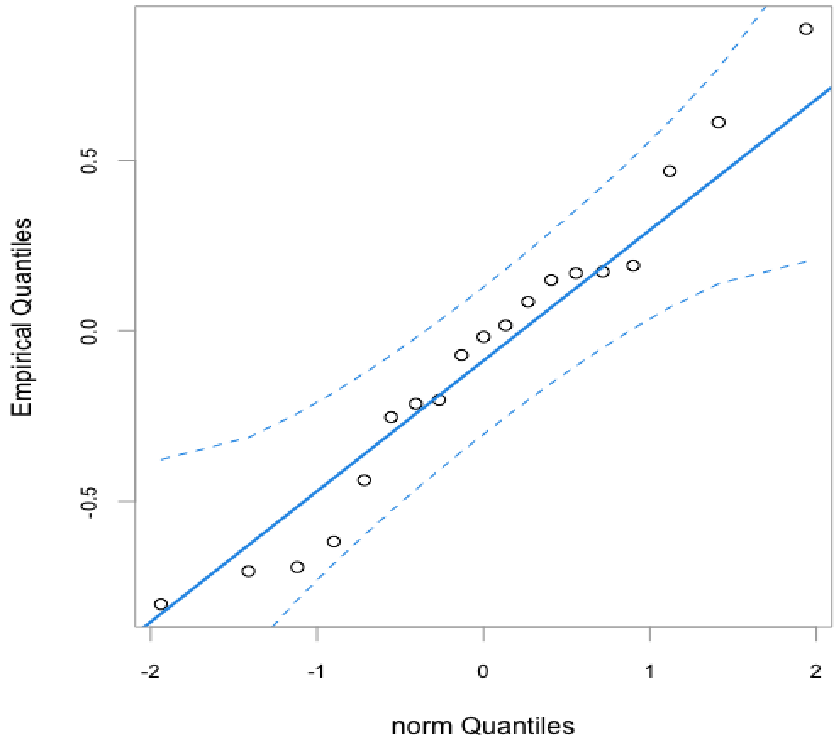

Additional File 3. Residual Plot for Generalized Linear Model of Distance Traveled by Weight

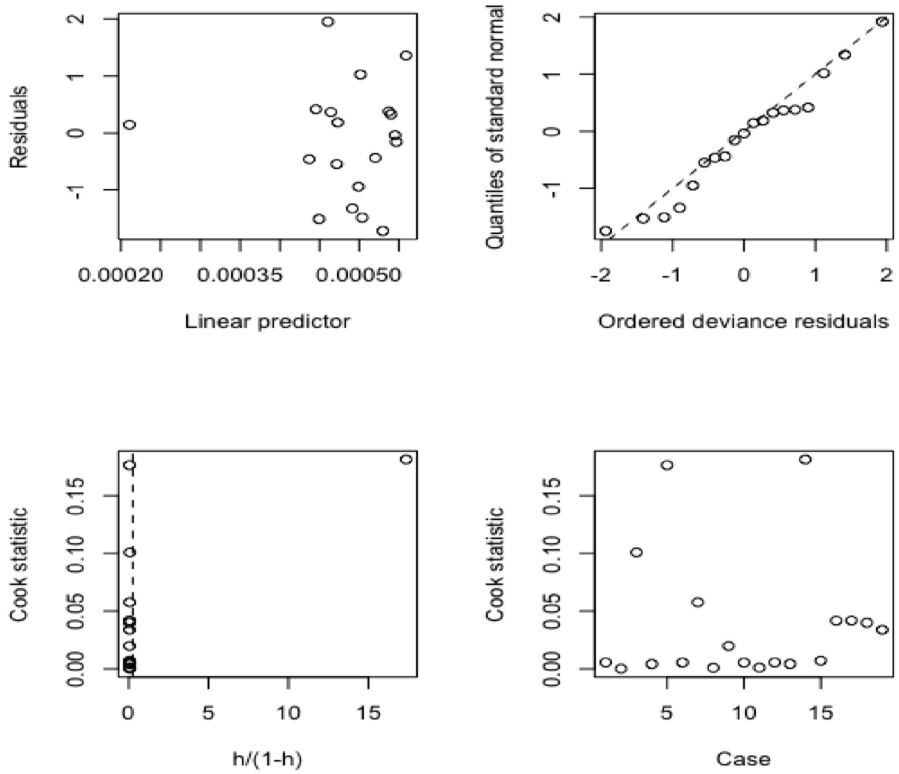

Additional File 4. Homogeneity of residuals

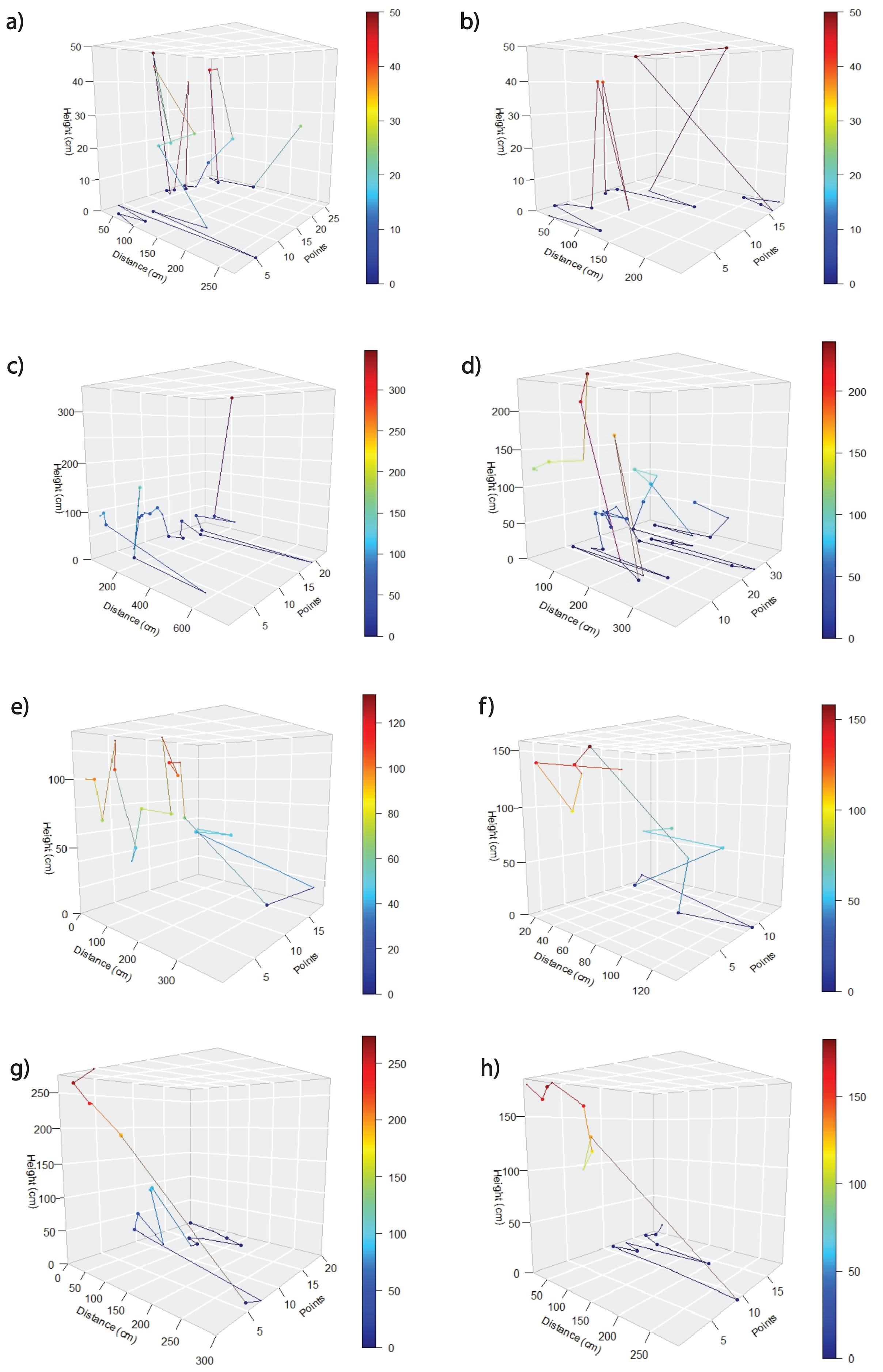

Additional File 5. Movements of individuals of *Phyllomedusa burmeisteri* during the four days of monitoring in open areas, showing three dimensions, distances traveled, height from ground and each point measured: a) individual PB3P2, b) individual PB1P1, c) individual PB2P1, d) individual PB3P1, e) PB4P1, f) individual PB5P1, g) individual PB6P1, h) individual PB7P1.

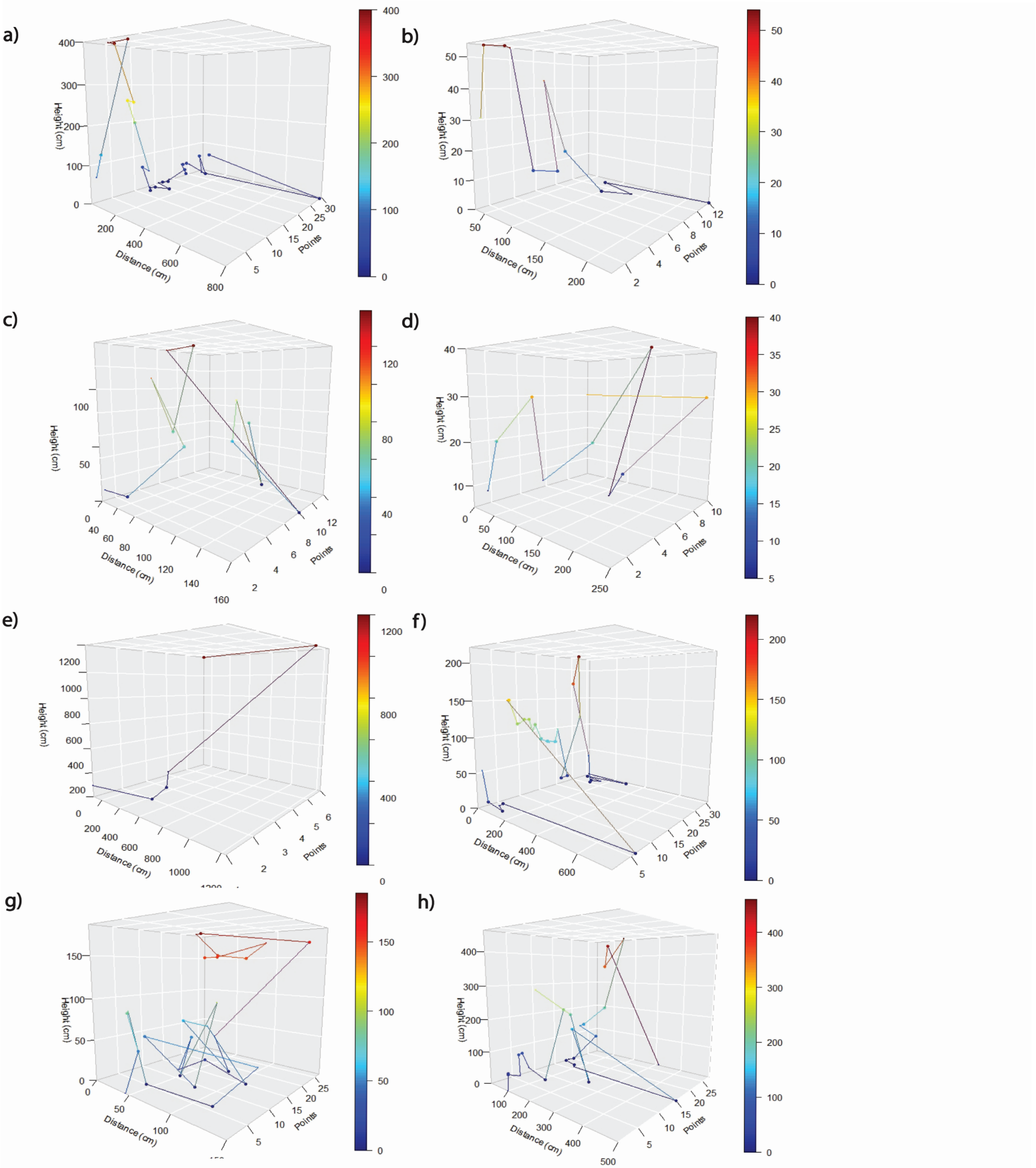

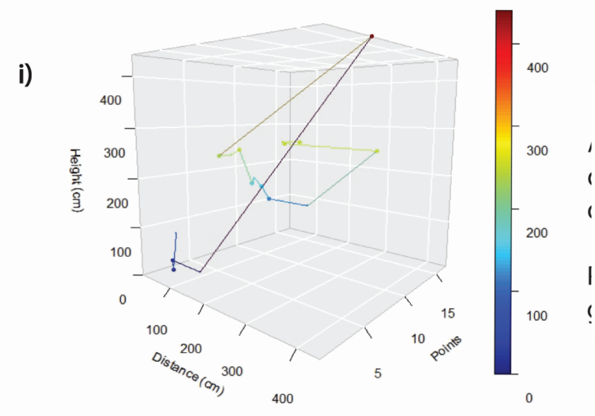

Additional file 6. Movements of individuals of *Phyllomedusa burmeisteri* during the four days of monitoring in forested areas, showing three dimensions, distances traveled, height from ground and each point measured: a) individual PB1MB, b) individual PB6MB, c) individual PB7MB, d) individual PB8MB, e) PB11MB, f) individual PB1V5, g) individual PB3V5, h) individual PB4V5, i)PB10MB.
